# An anti-MUC1-antibody–interleukin-2 fusion protein that activates resting NK cells to lysis of MUC1-positive tumour cells

**DOI:** 10.1038/sj.bjc.6601267

**Published:** 2003-09-09

**Authors:** C Heuser, M Ganser, A Hombach, H Brand, G Denton, F-G Hanisch, H Abken

**Affiliations:** 1 Lab. Tumorgenetik, Klinik I für Innere Medizin, Klinikum der Universität zu Köln, Joseph-Stelzmann-Str. 9, D-50931 Köln, Germany; 2 Cancer Research Laboratories, School of Pharmaceutical Sciences, University of Nottingham, Nottingham NG7 2RD, UK; 3 Zentrum für Biochemie und; 4 Zentrum für Molekulare Medizin, Medizinische Fakultät, Universität zu Köln, Joseph-Stelzmann-Str. 52, D-50931 Köln, Germany

**Keywords:** MUC1, scFv, antibody–cytokine fusion protein, immunocytokine, tumour antigen, natural killer cells, immunotherapy

## Abstract

MUC1 mucin is aberrantly glycosylated and overexpressed in a number of epithelial malignancies and is therefore a promising tumour-associated antigen for target-directed immunotherapy of a panel of malignant diseases. In MUC1-positive tumours, MHC class I expession is frequently downregulated and MUC1-specific cytotoxic T cells (CTLs) are either not available or in a state of anergy allowing tumour growth without limitation by CTL control. To activate lymphocytes and natural killer (NK) cells, we here generated an anti-MUC1-scFv-IL2 fusion protein (C595scFv-Fc-IL2) that contains the C595 single-chain antibody for MUC1 binding, the human IgG1 CH2CH3 domain for protein dimerisation, and interleukin-2 (IL2) for activation of immunological effector cells. The fusion protein binds to MUC1-derived peptides and to MUC1-positive tumour cells with the same specificity as does the C595 monoclonal antibody. Bound to MUC1, the C595scFv-Fc-IL2 fusion protein stimulates proliferation of human activated lymphocytes *in vitro*. Upon binding to MUC1-positive MCF7 breast carcinoma cells, moreover, the fusion protein activates resting NK cells to tumour cell lysis. These properties make the C595scFv-Fc-IL2 fusion protein a suitable candidate for the immunotherapy of MUC1-positive tumours.

MUC1 is a high molecular weight, type 1 transmembrane mucin with >50% carbohydrates O-linked to the protein core through serine and threonine. The unique extracellular mucin domain consists of variable numbers of tandemly repeated peptides (VNTR domain), each of them made up of 20 amino-acid sequences. The VNTR domain varies in length from 20 to over 100 repeats depending on the different alleles, exhibits a sequence polymorphism, and is characterised by differentiation-dependent profiles of complex glycans that control antigenicity and immunogenicity of the mucin (Hanisch and Müller, 2000). MUC1 is expressed on the luminal surfaces of glandular epithelial cells of respiratory, gastrointestinal and genitourinary tracts as well as of pancreatic and mammary ducts. Adenocarcinomas from these organs frequently overexpress and aberrantly glycosylate MUC1 leading to the suggestion that loss of MUC1 topological restriction and increased cellular expression in cancer cells may contribute to the malignant phenotype (Hilkens *et al*, 1992). As MUC1 is secreted by tumour cells, determination of the levels of MUC1 antigen in the blood has been exploited as a measure of tumour burden and changing levels as a reflection of the response to therapy (Berruti *et al*, 1994; Martoni *et al*, 1995).

Patients with MUC1-positive tumours develop both humoral and cellular immune responses against determinants on the MUC1 antigen from malignant cells as measured by *in vitro* parameters (Jerome *et al*, 1991; Rughetti *et al*, 1993; Kotera *et al*, 1994; Domenech *et al*, 1995; Hamanaka *et al*, 2003). Breast and pancreatic cancer patients may spontaneously develop a specific, MHC-independent cytotoxic T-cell response against epitopes in the protein core of tumour-associated MUC1 (Jerome *et al*, 1991); however, MHC class I and class II restricted cellular immune reactions against MUC1 protein were observed as well (Apostolopoulos *et al*, 1997; Hiltbold *et al*, 1998; Reddish *et al*, 1998; Brossart *et al*, 1999; Pietersz *et al*, 2000; Heukamp *et al*, 2001). Although anti-MUC1 responses are frequently detected in patients with advanced and progressing adenocarcinomas (Nakamura *et al*, 1998), activation of immunological effector cells is obviously not sufficient to protect against tumour progression *in vivo*. Natural B-cell responses to lactation- or tumour-associated glycoforms of the mucin, as detected in women after pregnancy or in some cancer patients, improve survival rates, but usually do not eliminate the MUC1+ tumour cells *in vivo*. Accordingly, enhancement of endogenous immune responses to MUC1 by active specific immunisation based on designed MUC1 vaccines is predicted to improve long-term survival of patients with MUC1+ adenocarcinomas. Recent evidence suggests that the induction of a strong anti-MUC1 response in cancer patients may require the activation of specific T helper cells via MHC class II presented peptide and glycopeptide epitopes, a process that is controlled by site-specific O-glycosylation (Vlad *et al*, 2002). CD4-mediated responses generated against MUC1 apparently do not fit the type 1 or 2 model (VanLith *et al*, 2002), and the involvement of natural killer cells (NK cells) in MUC1-based immunity has been demonstrated. The search for immunological strategies that allow an MUC1-specific and locally restricted activation of effector cells in the vicinity of the tumour could lead to alternative therapeutical tools.

We here made use of an immunocytokine to modulate specifically and locally the immune response. The concept of immunocytokines is based on fusion proteins composed of a cytokine for the activation of immune cells and of an antibody-derived binding domain for specific targeting of the cytokine (Lode *et al*, 1998). Strategies based on antifolate receptor scFv or anticarcinoembryonic antigen scFv were successfully evaluated in transgenic mouse models (Melani *et al*, 1998; Xu *et al*, 2000) proving the principal of the approach as promising. A panel of antibodies were raised against the tumour-associated form of the MUC1 antigen, particularly against the protein core motif (Price *et al*, 1997). The anti-MUC1 monoclonal antibody (mAb) C595 reacts with the peptide motif RPAP in the repeat sequence of the protein core (Price *et al*, 1990). C595 mAb has been used in immunoassays for the measurement of soluble MUC1 in breast cancer patients (Price *et al*, 1992; Dixon *et al*, 1993) and for *in vivo* immunoscintigraphy (Symonds *et al*, 1992; Perkins *et al*, 1993). The C595 mAb has been converted into a single-chain fragment of variable regions (scFv) with retained binding specificities (Denton *et al*, 1997). Utilising the C595 scFv, we generated an anti-MUC1-scFv-Fc-IL2 fusion protein that after binding to MUC1-positive tumour cells induces proliferation of peripheral blood T cells and mediates activation of resting NK cells to lysis of MUC1-positive tumour cells. Results presented here suggest the C595scFv-Fc-IL2 fusion protein as suitable immunocytokine for the specific immune modulation in the vicinity of MUC1-positive tumours.

## MATERIALS AND METHODS

### Cell lines

The human mammary carcinoma cell line T-47D (HTB-133, ATCC, Rockville, MD, USA) was cultured in RPMI 1640 medium supplemented with 2 mM L-glutamine (Sigma, Deisenhofen, Germany), 0.2 U ml^−1^ insulin (Novo Nordisk, Mainz, Germany) and 10% (v v^−1^) fetal calf serum (FCS, Biochrome KG, Berlin, Germany). The human mammary carcinoma cell line MCF7 (HTB-22, ATCC) and the human renal carcinoma line 293 T expressing the SV40 large T antigen (Pear *et al*, 1993) were maintained in DME medium with 10% (v/v) FCS. The Hodgkin's lymphoma-derived cell line L540 (Diehl *et al*, 1983) was cultured in RPMI 1640 medium, 2 mM L-glutamine, 10% (v /v) FCS.

### MUC1 glycoforms

Native MUC1 glycoforms were isolated from human milk fat globule membranes as described previously (Müller *et al*, 1997). A partially deglycosylated derivative of the lactation-associated glycoform was generated by treatment with trifluoromethane sulfonic acid for 30 min at 0°C (Müller *et al*, 1997).

### Chemical synthesis and purification of MUC1 peptides and glycopeptides

Glycopeptides A13, A9 and H4 corresponding to MUC1 tandem repeat peptides based on the AHG21 sequences AHGVTSAPDTRPAPGSTAPPA (A13, A9) and AHGVTSAPESR PAPGSTAPAA (H4) and carrying GalNAc at Thr/Ser10 (A13, H4) or at multiple sites (refer to Table 1

**Table 1:** (Glyco)peptides used in this study corresponding to sections of the human MUC1 repeat domain

**Designation**	**Structure**
TR3 (60-mer)	(PAPGSTAPPAHGVTSAPDTR)_3_
TR5 (100-mer)	(GVTSAPDTRPAPGSTAPPAH)_5_
TAP25	TAPPAHGVTSAPDTRPAPGSTAPPA
AHG21-AES	AHGVTSAP**ES**RPAPGSTAP**A**A
A13	AHGVTSAPDTRPAPGSTAPPA
H4	AHGVTSAP**ES**RPAPGSTAP**A**A
A11	HGVTSAPDTRPAPGSTAPPA
A9	AHGVTSAPDTRPAPGSTAPPA

O-Glycosylation sites are indicated as GalNAc or Gal - GalNAc, respectively. Sequence variations according to a polymorphism in the tandem repeat domain (Engelmann *et al*, 2001) are indicated by bold letters. TR5, 100-mer peptide, was kindly provided by Dr Olivera Finn, University of Pittsburgh, USA; TR3, 60-mer peptide, was obtained Dr. Jo Hilgers, previously at Academisch Ziekenhuis, Amsterdam, The Netherlands; TAP25 and AHG21-AES were synthesised in a local facility, and the glycopeptides were prepared by Professor Hans Paulsen, University of Hamburg, Germany.

) were chemically synthesised according to previously published protocols (Paulsen *et al*, 1995) and isolated successively on preparative and analytical reversed-phase columns on an HPLC workstation (System Gold, Beckman, München, Germany). The peptides TAP25 and AHG21-AES were chemically synthesised by a local facility at the Center of Biochemistry (Medical Faculty of the University of Cologne) and isolated on a preparative C18 reversed-phase column AQ20S11153OR (150 × 30 mm, YMC Europe GmbH). TR3 (60-mer) with repetitive PAP20 sequence was a kind gift of Dr Jo Hilgers (previously at Academisch Ziekenhuis, Amsterdam, The Netherlands) and TR5 (100-mer) with repetitive GVT20 was kindly provided by Dr. Olivera Finn (Department of Immunology, University of Pittsburgh, Pittsburgh, PA, USA).

### Monoclonal antibodies

C595 is a monoclonal antibody (IgG_3_, kappa light chain) with specificity for MUC1 urinary mucin (Price *et al*, 1990). The recombinant antibody fragment C595scFv was derived from the C595 antibody and is composed of the VH and VL variable regions joined via a (Gly_4_Ser)_3_ linker peptide (Denton *et al*, 1997).

### Construction and expression of the MUC1-specific fusion proteins C595scFv-Fc and C595scFv-Fc-IL2

The cDNA encoding the human IgG1 CH2/CH3 domain (Shu *et al*, 1993) as well as the IL2 cDNA, derived from Jurkat cells (TIB-152, ATCC), were terminally equipped by polymerase chain reaction PCR with *Bam*HI and *Xho*I restriction sites, respectively, using the following oligonucleotide primers:

hIgG-5′[5′-CTGAAGGATCCCGCCGAGCCCAAATCTCC TGACAAAACT-3′],

hIgG-Stop-3′[5′-GGCCTCGAGCTAGATCTTACCCGGAGACA GGGA-3′],

5′-hIL2,[5′-GATCAGGATCCGGTGATCAAAGCACCTACTTCAAGTTC TACA-3′],

3′-hIL2, [5′-TCAACTCGAGTCGACTCAAGTCAGTGTTG AGATGAT-3′] (restriction sites are underlined). The PCR products were ligated into the corresponding sites of the expression vector pRSV-HRS3scFv-*γ* that encodes a CD30-specific recombinant immunoreceptor (Hombach *et al*, 1998). Thereby, the Fc*ɛ*RI*γ* signalling domain is replaced by the cDNA coding for the human IgG1 constant domain and the IL2 cDNA, respectively. The DNA encoding the single-chain antibody fragment C595scFv was flanked by PCR with nucleotide sequences containing *Xba*I and *Bam*HI restriction sites (underlined), respectively, utilising the primer oligonucleotides VH5′, 5′-[GCGGCCCAGTCTAGAATGGCCCAG]-3′ and C595VL3′, 5′-[CGCACCTGGATCCGCCCGTTTCAGCTGCAG]-3′. The amplified C595 scFv DNA was subsequently inserted into the corresponding sites of pRSV-HRS3scFv-Fc DNA herewith replacing the HRS3 scFv domain. To construct the C595scFv-Fc-IL2 expression cassette, the C595scFv-Fc DNA was amplified by PCR and flanked with *Sna*BI and *Bgl*II restriction sites (underlined) utilising the plasmid DNA pRSV-C595scFv-Fc as template and the oligonucleotide primers Lkappa-5′ [5′-CTACGTACCATGGATTTTCAGGTGCAGAT TTTC-3′] and hIgG1-3′(BglII) [5′-CCCACCCAGATCTTTTTTACCCGGAGACA GGGAGAGGCTCTTCTG-3′]. The PCR product was ligated into the vector construct pRSV-HRS3scFvIL2 replacing the HRS3scFv binding domain. For expression of the recombinant anti-MUC1 fusion proteins, 293 T cells were transfected by calcium phosphate techniques with 10–20 *μ*g of the plasmid DNA pRSV-C595scFv-Fc and pRSV-C595scFvFc-IL2, respectively. After 48–72 h the cell culture supernatants were harvested and analysed for the presence of the fusion proteins.

### ELISA to detect the anti-MUC1 fusion proteins

Serial dilutions of the cell culture supernatant of transfected 293 T cells were incubated in microtitre plates coated with an anti-human IgG antibody (1 *μ*g ml^−1^, Southern Biotechnology, Birmingham, AL, USA). Bound fusion proteins were detected by a biotinylated anti-human IgG antibody (0.125 *μ*g ml^−1^, Southern Biotechnology) or, alternatively, by a biotin-labelled anti-human IL2 antibody (0.5 *μ*g ml^−1^, BD Biosciences/PharMingen). The assays were developed by means of a streptavidin-coupled peroxidase and ABTS substrate (both Roche Diagnostics, Mahnheim, Germany).

### Sodium dodecyl sulphate–polyacrylamide gel electrophoresis and Western blot analysis

The cell culture supernatant of transfected 293 T cells was electrophoretically separated by sodium dodecylsulphate polyacrylamide gel electrophoresis (SDS–PAGE) and blotted onto nitrocellulose membrane. The membrane was blocked with Roti®-Block (Roth GmbH, Karlsruhe, Germany) containing 5% milk powder and subsequently incubated with a horseradish peroxidase (HRP)-conjugated rabbit anti-human IgG antibody (1 : 20 000, Dako, Hamburg, Germany) or with a mouse anti-human IL2 antibody (1 : 250, Serotec, Düsseldorf, Germany), followed by incubation with a rabbit anti-mouse IgG-HRP conjugate (1 : 20 000, Dako), respectively. The blots were developed by means of the ECL™ detection reagent (Amersham Pharmacia Biotech, Freiburg, Germany).

### Antigen-binding assays

Binding of the C595scFv-Fc and C595scFv-Fc-IL2 fusion proteins was monitored by ELISA using partially deglycosylated MUC1 from human milk fat membranes (Müller *et al*, 1997) as well as a set of synthetic peptides and glycopeptides (Table 1).

Microtitre plates (maxi-sorp, Nunc, Roskilde, Denmark) were coated with the peptide antigens (10 *μ*g ml^−1^), partially deglycosylated MUC1 from human milk (100 *μ*g ml^−1^), BSM (100 *μ*g ml^−1^) and for control reason with an anti-human IgG antibody and an anti-mouse IgG1 antibody, respectively (both 1 *μ*g ml^−1^, Southern Biotechnology). After blocking the microtitre plates were incubated with cell culture supernatants containing the fusion proteins C595scFvFc and C595scFv-Fc-IL2, respectively, with the monoclonal antibody C595 (250 ng ml^−1^), and for control with a murine IgG1 monoclonal antibody (250 ng ml^−1^, Sigma). Bound fusion proteins and antibodies were detected with biotin-labelled anti-isotypic goat anti-human IgG and goat anti-mouse IgG antibody (both 125 ng ml^−1^, Southern Biotechnology). The assays were developed by means of a streptavidin-coupled peroxidase and ABTS substrate (both Roche Diagnostics).

To compare the antigen-binding capacity of the fusion proteins C595scFv-Fc and C595scFv-Fc-IL2, respectively, with that of the parental mAb C595, microtitre plates (maxi-sorp, Nunc) were coated with the peptide antigen TR5 (2.5 *μ*g ml^−1^), blocked and subsequently incubated with serial dilutions of cell culture supernatants containing the fusion proteins C595scFv-Fc and C595scFv-Fc-IL2, respectively, and dilutions of the parental mAb C595. The fusion proteins and the mAb bound to the microtitre plates were detected by biotin-labelled secondary antibodies as described above. Dilutions of supernatants and antibody resulting in half-maximal absorbance at 405 nm were applied to plates with immobilised TR5 antigen (2.5 *μ*g ml^−1^). Binding was assayed in the presence of increasing concentrations of soluble TR5 antigen (up to 20 *μ*g ml^−1^). The percentage of binding inhibition was calculated as follows:


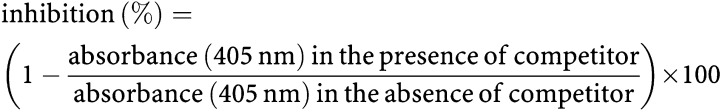


### Immunofluorescence and flow cytometry

Immunofluorescence assays were performed by incubation of T-47D cells (MUC1+/CD25−), 293 T cells (MUC1−/CD25−) and L540 cells (MUC1−/CD25+) with cell culture supernatant of transfected 293 T cells containing the fusion protein C595scFv-Fc and C595scFv-Fc-IL2, respectively, and as control with supernatant of mock-transfected 293 T cells. Alternatively, fusion proteins were incubated with resting peripheral blood lymphocytes (PBL) or lymphocytes activated with IL2 (400 U ml^−1^) and anti-CD3 antibody OKT3 (100 ng ml^−1^). Bound fusion proteins were detected by an FITC-labelled F(ab′)_2_ anti-human IgG antibody (2 *μ*g ml^−1^, Southern Biotechnology). The IL2 receptor (*α*-chain) was detected by incubation of PBL with an FITC-conjugated anti-CD25 antibody (clone B1.49.9, Coulter-Immunotech Diagnostics, Hamburg, Germany). Immunofluorescence assays were analysed utilising a FACScan™ cytofluorometer equipped with the Cellquest™ analysis software (Becton Dickinson, Mountain View, CA, USA).

### Proliferation assays

Peripheral blood lymphocytes from healthy donors were isolated from peripheral blood by density centrifugation and cultured for 24 to 48 h in RPMI 1640 medium supplemented with 10% (v /v) FCS, IL2 (400 U ml^−1^), and anti-CD3 mAb OKT3 (100 ng ml^−1^). Activated lymphocytes were washed twice and cultured for additional 48 h in RPMI 1640 medium containing 10% (v /v) FCS, IL2 (400 U ml^−1^). Peripheral blood lymphocytes were washed twice and cultured for 3 h in the absence of IL2. Subsequently 2.5 × 10^5^ PBL per well were cultured in the presence of serial dilutions of cell culture supernatant containing the fusion proteins C595scFv-Fc-IL2 or C595scFv-Fc and for control IL2 (2000 U ml^−1^), respectively. After 72 h, lymphocyte proliferation was quantified by means of the ‘XTT cell proliferation kit’ (Roche Diagnostics) according to the manufacturer's recommendations.

Alternatively, activated PBL were cultured in the presence of fusion protein bound to immobilised antigen. Microtitre plates (maxi-sorp, Nunc) were coated with increasing amounts of MUC1 antigen, TR5 glycopeptide, and asialo-BSM, respectively (up to 100 *μ*g ml^−1^). After blocking, the coated wells were incubated with cell culture supernatants containing the fusion proteins C595scFv-Fc and C595scFv-Fc-IL2. The plates were washed three times with PBS and lymphocytes (2.5 × 10^5^ per well) were added. After culture for 72 h, proliferation was monitored utilising ‘XTT cell proliferation kit’ (Roche Diagnostics).

Alternatively, polystyrene-coated ferric oxide beads M-280 (tosylated, Dynal, Hamburg, Germany) were covalently coated with TR5 peptide antigen and for control reason with human serum albumin (following the manufacturer's recommendations). The beads were incubated with cell culture supernatants of transfected 293 T cells containing the fusion proteins C595scFv-Fc and C595scFv-Fc-IL2, respectively, and supernatant of untransfected 293 T cells. Specific binding of the fusion proteins to TR5-coated beads was monitored by flow cytometry utilising a FITC-labelled F(ab')_2_ anti-human IgG antibody (2 *μ*g ml^−1^, Southern Biotechnology). After three times of washing, serial dilutions of coated beads were seeded onto 96-well microtiter plates (15±10^5^–0.6±10^5^ beads/well) and activated lymphocytes (2 × 10^5^ cells–well) were added. After 72 h, proliferation was determined as described.

### Cytotoxicity assay

Tumour cell viability was monitored by an XTT-based colorimetric assay according to Jost *et al*. (1992). Briefly, PBL from healthy donors were isolated by density centrifugation and NK cells were negatively enriched by magnetic activated cell sorting (MACS) utilising the ‘NK Cell Isolation Kit’ (Miltenyi Biotec, Bergisch Gladbach, Germany). MUC-1-positive MCF7 cells or T47D cells were incubated with cell culture supernatants containing C595scFv-Fc and C595scFv-Fc-IL2 fusion protein, respectively, and for control with medium with and without IL2 (400 U ml^−1^). After four times of washing, coated target cells were seeded onto round-bottomed microtiter plates (5 × 10^4^ cells per well) together with resting NK cells (1 × 10^5^ per well). In a second set of experiments, untreated tumour cells (5 × 10^4^ per well) were cocultured with NK cells (1 × 10^5^ per well) in the presence of the soluble fusion protein C595scFv-Fc-IL2 and IL2 (400 U ml^−1^), respectively. After 48 h, the XTT reagent (1 mg ml^−1^, ‘Cell Proliferation Kit II’, Roche Diagnostics) was added to the cells and incubated for 60–90 min at 37°C. Metabolisation of XTT to a formazan was monitored colorimetrically at an adsorbance wavelength of 450 nm and a reference wavelength of 650 nm. Maximal reduction of XTT was determined as the mean of three wells containing tumor cells only, and background was determined as the mean of three wells containing RPMI 1640 medium, 10% (v v^−1^) FCS. The formation of formazan due to the effector cells was determined from triplicate wells containing NK cells only. The percentage of viable tumour cells was calculated as follows:


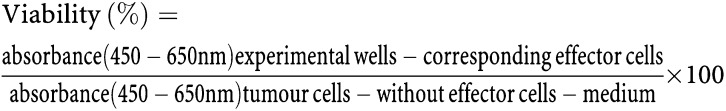


## RESULTS

### Expression of the recombinant fusion proteins C595scFv-Fc and C595scFv-Fc-IL2

**Figure 1:**
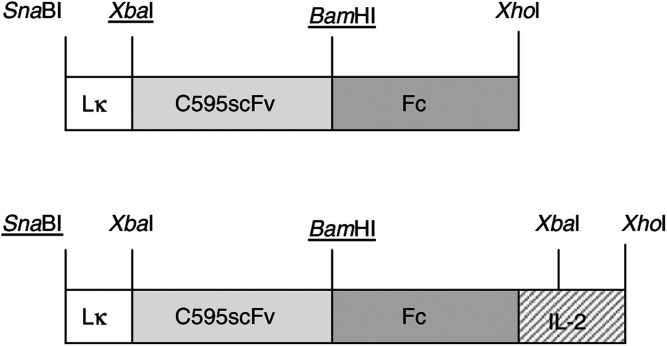
Expression cassettes coding for the recombinant anti-MUC1 fusion proteins C595scFv-Fc and C595scFv-Fc-IL2. The expression cassettes were generated as described in Materials and Methods. Lk: leader sequence of the Ig *κ* chain; C595scFv: anti-MUC1 scFv; Fc: IgG1 CH2CH3. The expression cassettes are driven by the RSV LTR.

**Figure 2:**
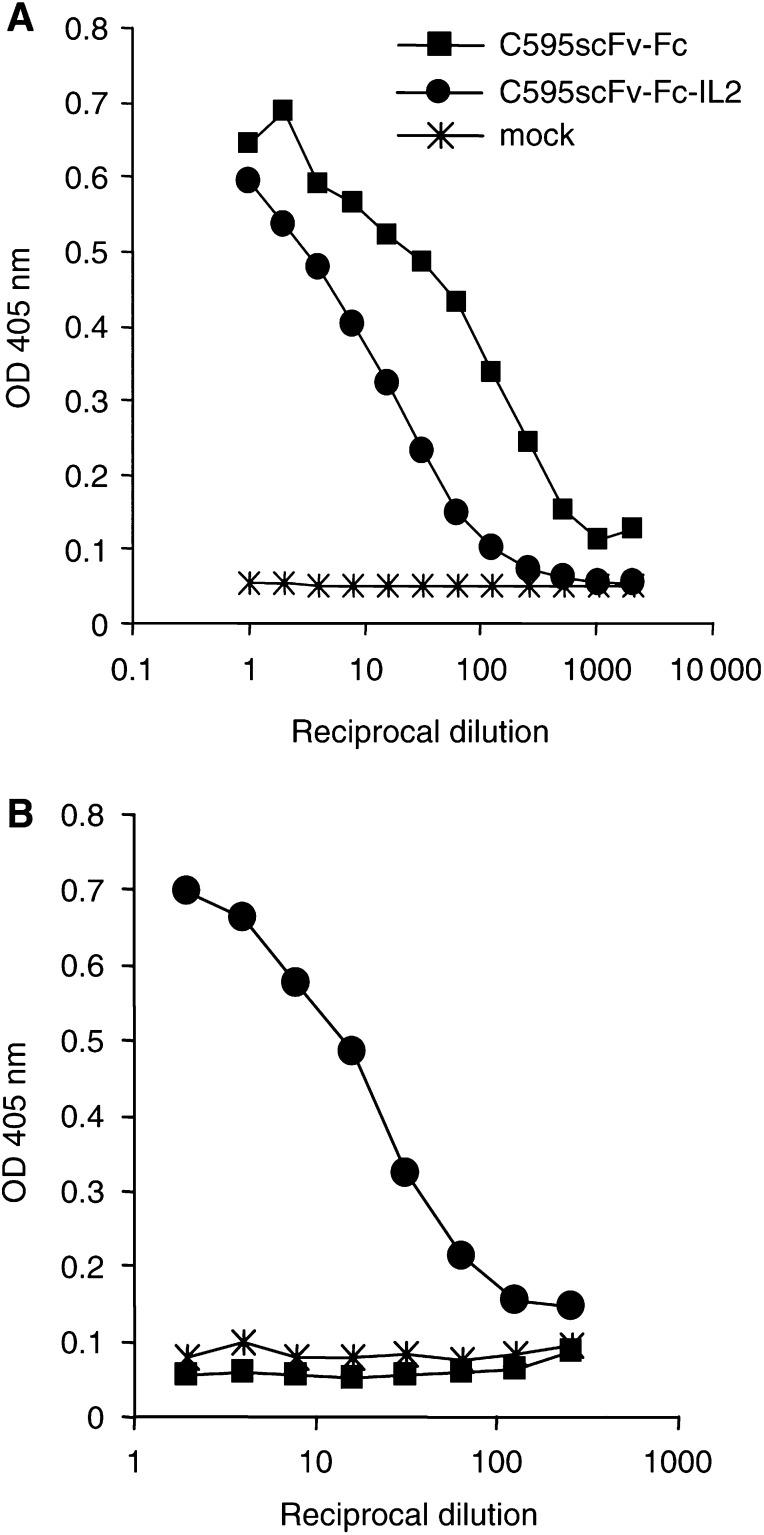
Detection of the fusion proteins C595scFv-Fc and C595scFv-Fc-IL2 in the supernatants of transfected 293 T cells. Serial dilutions of culture supernatants from 293 T cells transfected with pRSV-C595scFv-Fc (▪), pRSV-C595scFv-Fc-IL2 (•) and, as control, from mock-transfected cells (*), respectively, were incubated in microtitre plates coated with an anti-human IgG antibody. Bound fusion proteins were detected by a biotin-labelled anti-human IgG (**A**) or anti-human IL2 (**B**) antibody.

**Figure 3:**
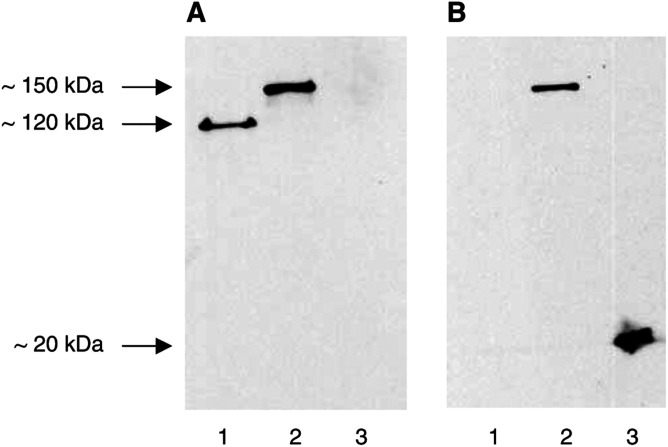
The fusion proteins C595scFv-Fc and C595scFv-Fc-IL2 are expressed as homodimers. Cell culture supernatants from 293 T cells transfected with pRSV-C595scFv-Fc DNA (lane 1) or with pRSV-C595scFv-Fc-IL2 DNA (lane 2) as well as recombinant human IL2 (1000 U, lane 3) were electrophoretically separated under nonreducing conditions, blotted onto nitrocellulose membrane and probed with an anti-human IgG antibody (**A**) and an anti-human IL2 antibody (**B**). The calculated molecular weight of the monomeric form of the fusion protein C595scFv-Fc is 60 kDa, of the C595scFv-Fc-IL2 protein is 75 kDa.

We generated the expression constructs pRSV-C595scFv-Fc and pRSV-C595scFv-Fc-IL2 that code for fusion proteins consisting of the anti-MUC1 scFv C595 and the human IgG1 CH2CH3 domain (Fc domain) with or without linked IL2, respectively (Figure 1

). After transient transfection, 293 T cells secrete the fusion proteins into the cell culture medium as monitored by ELISA (Figure 2

). Whereas both proteins are captured by the anti-human IgG antibody directed against the Fc domain of the fusion proteins, only the C595scFv-Fc-IL2 immunocytokine is detected by the anti-human IL2 antibody. Western blot analysis revealed that the C595scFv-Fc fusion protein migrates at about 120 kDa and the C595scFv-Fc-IL2 fusion protein at about 150 kDa under nonreducing conditions (Figure 3A

) corresponding to the homodimeric forms of the respective fusion proteins. Western blot analysis moreover confirmed the IL2 cytokine as part of the C595scFv-Fc-IL2 fusion protein (Figure 3B).

### Fusion proteins bind specifically to MUC1

**Figure 4:**
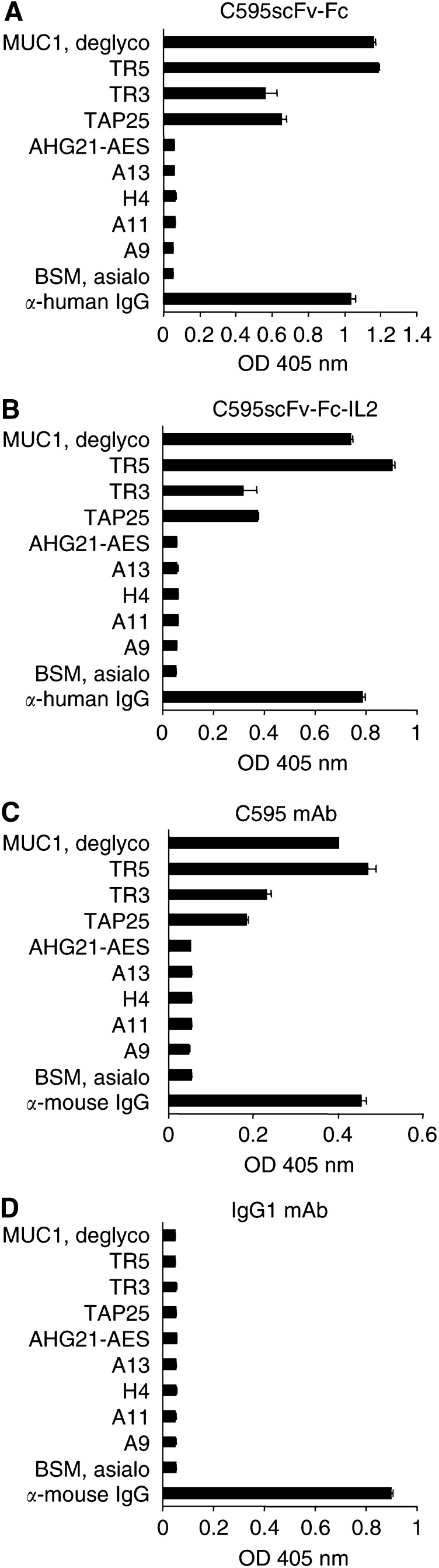
Antigen binding profile of the recombinant fusion proteins C595scFv-Fc and C595scFv-Fc-IL2. Microtitre plates were coated with a set of MUC1-related (glyco-) peptides (10 *μ*g ml^−1^) corresponding to mono- and oligorepeats and containing the RPAP motif (see Table 1), partially deglycosylated MUC1 antigen (100 *μ*g ml^−1^), and, as control, BSM (100 *μ*g ml^−1^), an anti-human IgG antibody (1 *μ*g ml^−1^) and an anti-mouse IgG antibody (1 *μ*g ml^−1^), respectively. The coated wells were incubated with cell culture supernatants containing the fusion proteins C595scFv-Fc (**A**) and C595scFv-Fc-IL2 (**B**), respectively, and as control with the mAb C595 (**C**) and a mouse IgG1 control antibody (**D**) (both 0.25 *μ*g ml^−1^). Bound fusion proteins and antibodies were detected by a biotin-labelled anti-human IgG mAb (**A** and **B**) or a biotin labelled anti-mouse IgG mAb (**C** and **D**). The assay was performed in triplicate; data are presented as the mean±s.e.m.

The fusion proteins C595scFv-Fc and C595scFv-Fc-IL2 and the mAb C595 were tested by ELISA for binding to partially deglycosylated MUC1 antigen from human milk fat membranes as well as to a set of MUC1 glycopeptides corresponding to parts of the repeat domain and containing the RPAP motif (Table 1). As shown in Figure 4

, the binding patterns of the fusion proteins to the panel of MUC1-derived antigens are similar to that of the monoclonal antibody C595 indicating that the C595 scFv domain in the fusion proteins conserved the antigen-binding profile of the parental C595 mAb. The profile is characterised by (i) binding to DTRPAP-containing repeat peptides (Table 1), (ii) no cross-reactivity to ESRPAP-containing repeat peptides (AHG21-AES, H4), which represent a known sequence polymorphism in the tandem repeat domain of the mucin (Hanisch and Müller, 2000; Engelmann *et al*, 2001), (iii) stronger binding to oligorepeats (TR3 and TR5) and native MUC1 (MUC1deglyco) than to monorepeats (TAP25), and (iv) inhibition of antibody binding due to O-glycosylation of the peptides (A13, A11, A9).

**Figure 5:**
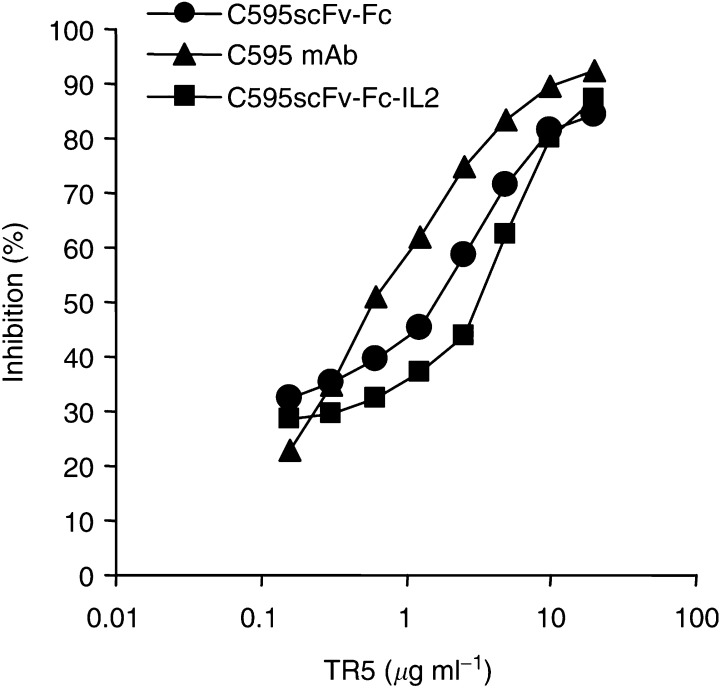
Binding-inhibition assay. Microtitre plates coated with the peptide antigen TR5 (2.5 *μ*g ml^−1^) were incubated with dilutions of C595scFv-Fc (•), C595scFv-Fc-IL2 (▪), and C595 mAb (▴), respectively, that result in half-maximal binding. The binding to immobilized TR5 antigen was specifically competed by addition of increasing amounts of soluble TR5 antigen. Bound fusion proteins were detected by the biotin-labelled anti-human IgG antibody; bound C595 mAb was detected by the biotinylated anti-mouse IgG antibody. The percentage of inhibition was determined as described in ‘Material and Methods’.

We furthermore compared the binding properties of the recombinant proteins C595scFv-Fc and C595scFv-Fc-IL2, respectively, to that of the mAb C595 by competition assays utilising dilutions of the fusion proteins and of mAb C595, respectively, that lead to half-maximal binding. Binding to immobilised TR5 peptide was assayed in the presence of increasing amounts of soluble TR5 peptide. As summarised in Figure 5

, binding of both fusion proteins C595scFv-Fc and C595scFv-Fc-IL2 is titrated out by increasing concentrations of soluble TR5 peptide. The apparent antigen-binding affinities of the recombinant fusion proteins, however, are reduced by a factor of 2–3 compared to that of the mAb C595 in this assay.

**Figure 6:**
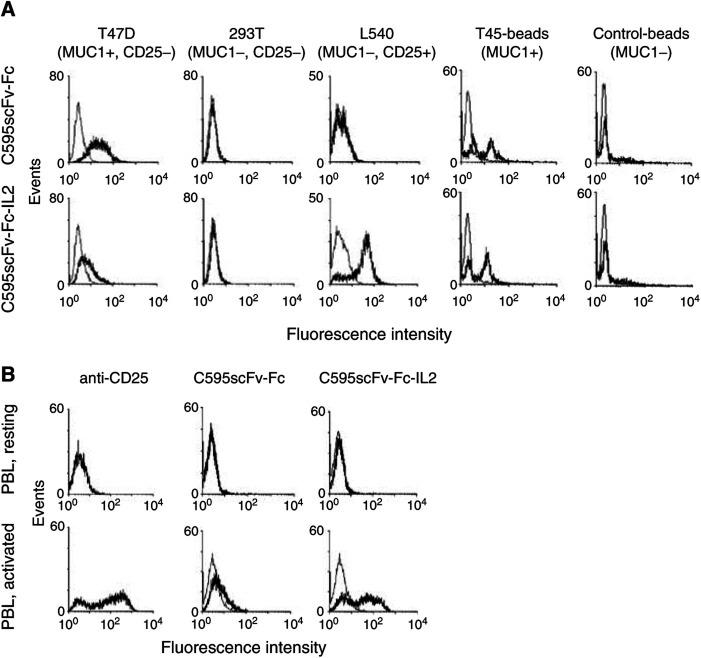
Fusion proteins C595scFv-Fc and C595scFv-Fc-IL2 bind specifically to MUC1 positive tumour cells and TR5 peptide-coated beads. Cell culture supernatants from transfected 293 T cells containing the C595scFv-Fc and C595scFv-Fc-IL2 fusion protein (thick lines), respectively, and, as control, supernatant from 293 T cells transfected with an irrelevant DNA (thin lines), were incubated (**A**) with T-47D cells (MUC1+/CD25−), 293 T cells (MUC1−/CD25−), L540 cells (MUC1−/CD25+), TR5 peptide-coated beads or, as control, with human serum albumin-coated beads, and (**B**) with quiescent lymphocytes from the peripheral blood (PBL) or with PBL activated by preincubation with the anti-CD3 mAb OKT3 plus IL2. Bound fusion proteins were detected by an FITC-labelled anti-human IgG antibody. Expression of the IL2 receptor CD25 was monitored by incubation with a FITC-conjugated anti-CD25 mAb (anti-CD25).

The fusion proteins were furthermore assayed by flow cytometry for binding to MUC1-expressing tumour cells. As demonstrated in Figure 6A

, both fusion proteins C595scFv-Fc and C595scFv-Fc-IL2 bind to cells of the MUC1-positive line T-47D but not to cells of the MUC1-negative 293 T line. The fusion proteins bind to polystyrene ferric oxide beads coated with TR5 peptide, but do not bind to beads coated with human serum albumin as control. C595scFv-Fc-IL2 protein, moreover, binds to the IL2 receptor (CD25) constitutively expressed on the surface of MUC1-negative L540 cells. Binding of C595scFv-Fc-IL2 to the IL2 receptor is specific because (i) the C595scFv-Fc fusion protein that lacks the IL2 domain does not bind to MUC1^−^ CD25^+^ L540 cells (Figure 6A), and (ii) C595scFv-Fc-IL2 binds to activated lymphocytes with increased CD25 expression after stimulation with the anti-CD3 antibody OKT3 plus IL2, but does not bind to resting lymphocytes that do not express the IL2 receptor (Figure 6B). As control, the C595scFv-Fc protein without IL2 domain does not bind to resting lymphocytes, but binds weakly via the C595 scFv domain to activated T cells that express Muc1 (Agrawal *et al*, 1998). Taken together, the fusion proteins C595scFv-Fc and C595scFv-Fc-IL2 specifically bind to MUC1-positive tumour cells via the anti-MUC1 scFv domain, the C595scFv-Fc-IL2 protein binds in addition via IL2 to CD25^+^, activated lymphocytes *in vitro*.

### C595scFv-Fc-IL2 fusion protein induces proliferation of activated lymphocytes

**Figure 7:**
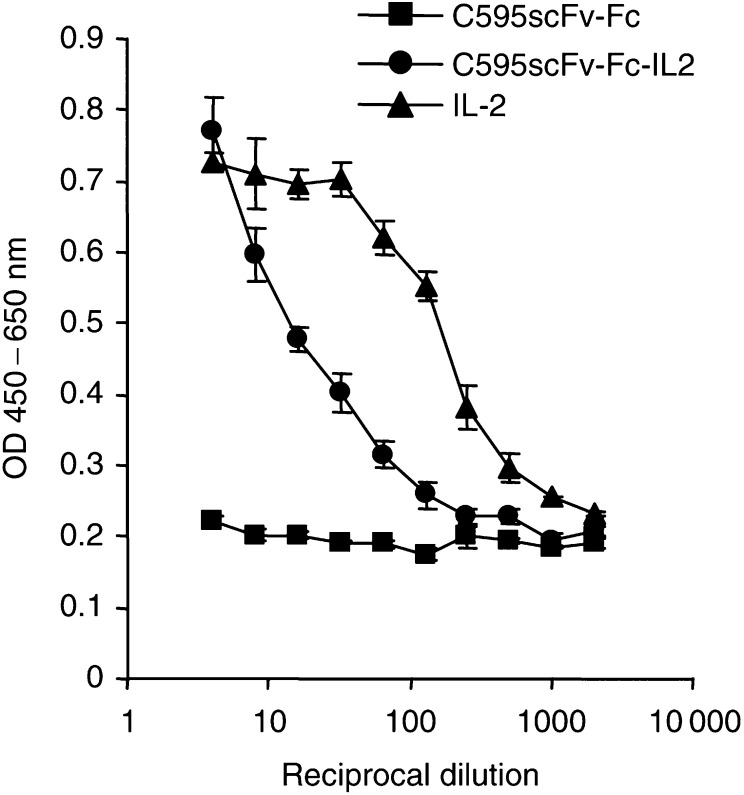
Activated lymphocytes proliferate in the presence of soluble C595scFv-Fc-IL2 fusion protein. Lymphocytes from the peripheral blood (2.5 × 10^5^ cells per well) were activated by incubation with the anti-CD3 mAb OKT3 plus IL2 and cultured in the presence of cell culture supernatants containing the fusion proteins C595scFv-Fc-IL2 (•), C595scFv-Fc (▪), and as control IL2 (▴) (2 000 U ml^−1^), respectively. After 72 h, lymphocyte proliferation was quantified by means of the ‘XTT cell proliferation kit’ (Roche Diagnostics). The assays were performed in triplicate; data are presented as mean±s.e.m.

We assessed the IL2 bioactivity of the C595scFv-Fc-IL2 fusion protein by monitoring the induction of proliferation of activated lymphocytes. As shown in Figure 7

the C595scFv-Fc-IL2 protein stimulates proliferation of activated lymphocytes in a dose-dependent manner, whereas the C595scFv-Fc protein that lacks the IL2 domain does not. Incubation of activated lymphocytes with IL2 served as control. Resting T cells, in contrast, are not activated to proliferate in the presence of the C595scFv-Fc-IL2 fusion protein (data not shown).

**Figure 8:**
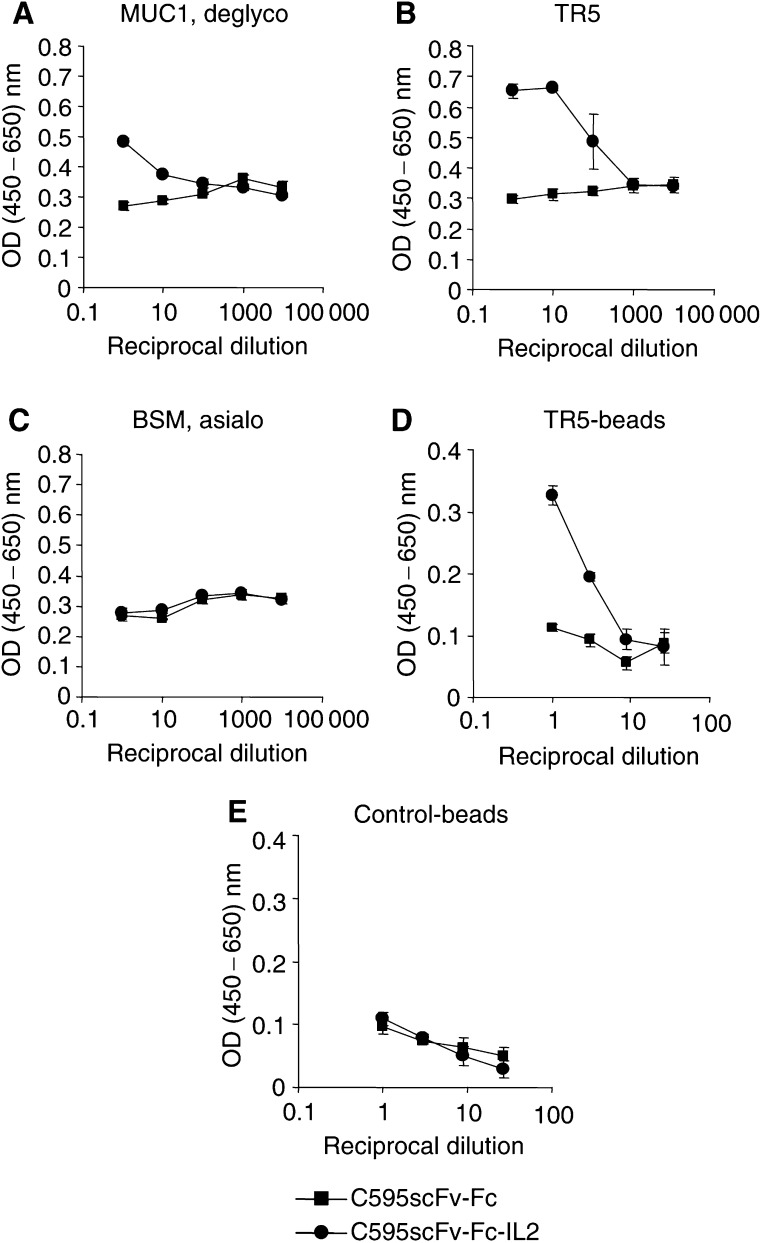
C595scFv-Fc-IL2 fusion protein bound to MUC1 antigen stimulates proliferation of activated lymphocytes. Micotitre immunoplates were coated with increasing amounts of deglycosylated MUC1 (**A**), TR5 peptide (**B**), and asialo-BSM (**C**), respectively, and subsequently incubated with cell culture supernatants containing the fusion proteins C595scFv-Fc (▪) and C595scFv-Fc-IL2 (•), respectively. After extensive washing, activated lymphocytes (2.5 × 10^5^ per well) were incubated in coated plates for 72 h. In a second set of experiments, beads coated with TR5 peptide antigen (**D**) and, as control, coated with human serum albumin (**E**) were incubated with culture supernatant containing the fusion protein C595scFv-Fc or C595scFv-Fc-IL2, respectively, washed extensively and incubated in serial dilutions (15 × 10^5^–0.6 × 10^5^ beads per well) with activated lymphocytes (2.5 × 10^5^ per well). After 72 h, lymphocyte proliferation was quantified by means of the ‘XTT cell proliferation kit’. The assays were performed in triplicate; data are presented as the mean±s.e.m.

To assay whether the C595scFv-Fc-IL2 fusion protein retains its IL2 bioactivity after binding to MUC1 antigen, we incubated C595scFv-Fc-IL2 protein, and C595scFv-Fc protein as control, on plastic surfaces coated with MUC1 or TR5 peptide, respectively. Bound to MUC1 and TR5 antigen, respectively, the C595scFv-Fc-IL2 protein stimulates proliferation of activated lymphocytes in a dose-dependent manner, whereas no lymphocyte proliferation was detected in the presence of the bound C595scFv-Fc fusion protein (Figure 8A, B

). As control, plates coated with asialo-BSM and incubated with the C595scFv-Fc-IL2 fusion protein did not induce proliferation of activated lymphocytes (Figure 8C).

In a second assay, tosylated ferric oxide beads were coated with TR5 peptide, or with human serum albumin as a control, and subsequently incubated with the fusion proteins C595scFv-Fc-IL2 and C595scFv-Fc, respectively. Binding of the fusion proteins on TR5-coated beads was confirmed by FACS analysis (Figure 6A). As summarised in Figure 8D, C595scFv-Fc-IL2 fusion protein bound to TR5-coated beads stimulates proliferation of activated lymphocytes in a dose-dependent manner, whereas C595scFv-Fc control protein does not. As control, beads coated with human serum albumin and incubated with the fusion proteins did not induce proliferation of activated lymphocytes (Figure 8E).

### C595scFv-Fc-IL2 fusion protein induces resting NK cells to lysis of MUC1-positive tumour cells

**Figure 9:**
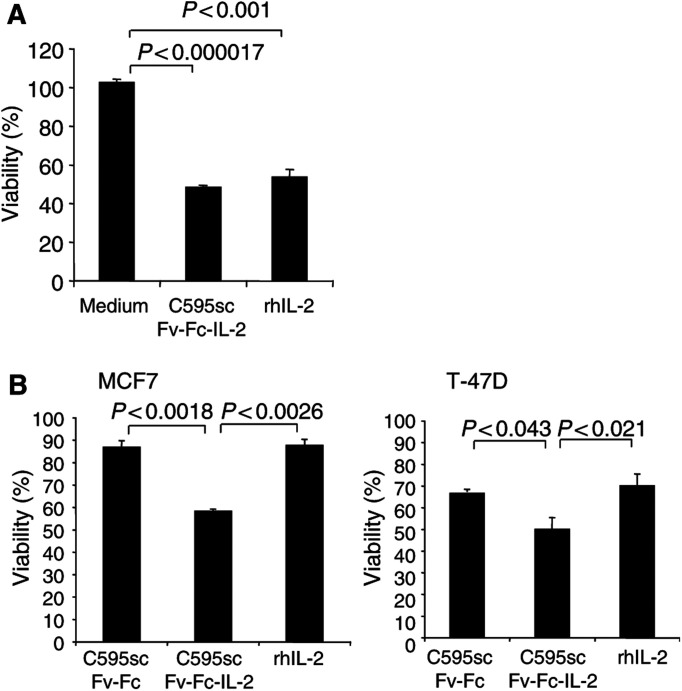
The fusion protein C595scFv-Fc-IL2 activates resting NK cells to tumour cell lysis (**A**). MCF7 cells (MUC1^+^) (5 × 10^4^ cells per well) were cocultured with resting NK cells (1x10^5^ cells per well) in the presence of C595scFv-Fc-IL2 fusion protein or IL2 (400 U ml^−1^). As control, MCF7 cells were cocultured with NK cells in cell culture medium without additives. (**B**) MCF7 and T-47D tumour cells (MUC1^+^) were preincubated with cell culture supernatants containing C595scFv-Fc, C595scFv-Fc-IL2, or IL2 (400 U ml^−1^), respectively. After four rounds of washings, cells were seeded into round-bottom microtiter plates (5 × 10^4^ cells per well) and cocultured with resting NK cells (1 × 10^5^ cells per well). The viability of MCF7 and T-47D cells was determined by an XTT-based viability assay as described in Material and Methods. The assays were performed in triplicate; data are presented as the mean±s.e.m. Statistical significance was determined utilising Student's *t*-test.

We assayed the bioactivitiy of the C595scFv-Fc-IL2 fusion protein bound to MUC1-positive target cells by monitoring the activation of resting NK cells to lyse target cells. MUC1-positive MCF7 tumour cells were incubated with C595scFv-Fc-IL2 fusion protein and IL2, respectively, and cocultured with resting NK cells. Tumour cell viability was monitored by an XTT-based colorimetric assay. As shown in Figure 9A

, C595scFv-Fc-IL2 protein induced resting NK cells to lysis of MCF7 cells as does IL2 indicating that the fusion protein is capable to activate resting NK cells.

In a second assay, MCF7 cells were preincubated with C595scFv-Fc-IL2, C595scFv-Fc, and IL2 as control, respectively, washed intensively, and subsequently coincubated with resting NK cells. As shown in Figure 9B, MCF7 cells coated with C595scFv-Fc-IL2 protein were significantly lysed by NK cells compared to preincubation with C595scFv-Fc protein without the IL2 domain. Soluble IL2 not bound to MCF7 cells and washed out did not induce NK-cell-mediated cytolysis indicating that soluble IL2 is substantially eliminated by repeated washings prior to coincubation with NK cells. Essentially, the same results were obtained when using Muc1^+^ T-47D cells in the assay (Figure 9B). This set of experiments demonstrates that the C595scFv-Fc-IL2 fusion protein bound to MUC1-positive tumour cells activates resting NK cells to tumour cell lysis.

## DISCUSSION

The purpose of this study was to engineer a novel immunotherapeutic reagent that is directed to MUC1-positive tumour cells and activates effector responses of NK cells. Activation of resting NK cells in particular is expected to be of significance considering recent evidences that NK cells may contribute to MUC1 immunity and that the MUC1 directed immune response does not fit the type 1 or type 2 model (VanLith *et al*, 2002). In the light of these findings the insufficient outcomes of multiple efforts to stimulate an MUC1-specific CD4^+^ or CD8^+^ T-cell response may find an explanation.

The anti-MUC1 scFv-Fc-IL2 fusion protein harbours the C595 scFv domain for targeting IL2 to MUC1-positive tumours. C595 scFv represents, to our best knowledge, the only example of a successful attempt to generate high-affinity anti-DTR MUC1-specific scFv from phage libraries or from established hybridoma clones. This antibody defines a peptide motif more upstream of the DTR sequence (RPAP). We here demonstrate that the conservative replacement of Asp-Thr within the sequence PDTRPAP by Glu-Ser has a severe negative impact on the binding of both, the parental antibody C595 and the scFv-Fc-IL2 fusion protein, which indicates a contribution of these two residues to the epitope structure of the C595 antibody. The replacement of DT by ES represents a sequence polymorphism found with a high incidence in all repeat domains of human MUC1, which were analysed on the level of genomic DNA (Engelmann *et al*, 2001). The C595 scFv binds to a panel of mammary and bladder carcinoma biopsies but not to benign tissues like breast ducts (Denton *et al*, 1997) making the scFv suitable for specific tumour cell binding.

The C595scFv-Fc-IL2 fusion protein is expressed as homodimer (cf. Figure 3) that is likely to be formed by disulphide bonds between the IgG1 Fc domains incorporated into the fusion protein between the scFv and IL2 moiety. Utilising a panel of MUC1 mucins and peptides, we here demonstrate that the C595scFv-Fc-IL2 fusion protein exhibits the same binding specificity as the C595 mAb (Figure 4), however, with slightly reduced avidity compared to that of the mAb C595 (Figure 5). The C595scFv-Fc-IL2 fusion protein binds specifically to the IL2 receptor on activated lymphocytes, but not to resting lymphocytes that do not express the IL2 receptor (Figure 6B). C595scFv-Fc weakly binds to activated lymphocytes, most likely via the C595scFv domain to Muc1 expressed on activated T cells (Agrawal *et al*, 1998). Although C595scFv-Fc-IL2 binds much stronger to activated lymphocytes via CD25, binding to activated T cells and dendritic cells via Muc1 cannot be excluded. Noteworthy, binding to resting T cells was not observed (cf. Figure 6B).

C595scFv-Fc-IL2 fusion protein stimulates proliferation of activated lymphocytes from the peripheral blood in a dose-dependent manner, whereas the C595scFv-Fc protein that lacks the IL2 domain does not. The IL2 activity of the fusion protein to induce proliferation of activated T cells is still conserved after binding to MUC1 mucin (cf. Figure 8). Resting T cells that do not express the IL2 receptor (CD25) are not activated by the fusion protein. Resting NK cells, in contrast, are activated by the C595scFv-Fc-IL2 fusion protein to tumour cell lysis (cf. Figure 9). These results are of major significance because (i) the fusion protein is designed to act locally in the near vicinity of the tumour after binding to MUC1-positive tumour cells and (ii) the fusion protein activates resting NK cells and preactivated T cells thereby recruiting both compartments of the cellular immune response. Noteworthy, resting T cells are not activated thereby avoiding an immune response of T cells that are not activated in the tumour environment and may be of nontumour-related specificities (not shown).

Strategies during the last years to improve the cellular immune responses against MUC1 protein include: (i) conjugation of MUC1 peptides with KLH or mannan (Karanikas *et al*, 1997; Gilewski *et al*, 2000), (ii) coexpression of MUC1 with CD80 (B7.1) in tumour cells to provide costimulatory signals to the immune system (Smith *et al*, 1999), (iii) fusing MUC1-positive tumour cells with antigen-presenting cells or pulsing dendritic cells with MUC1-derived peptides (Brossart *et al*, 2000; Gong *et al*, 2000), (iv) application of MUC1 together with Th1 cytokines (Acres *et al*, 2000; Carr-Brendel *et al*, 2000; Lees *et al*, 2000; Mukherjee *et al*, 2003), and (v) use of a BCG-MUC1-IL2 fusion protein (He *et al*, 2002). The latter fusion protein consists of the BCG antigen signal sequence, the MUC1 VNTR core peptide, and IL2. Immunisation of SCID/hu-PBL mice with the fusion protein resulted in increased MUC1-specific cellular immunity suggesting that combined application of MUC1 peptide and IL2 may mediate an effective immunity against MUC1-positive cancer. As far as clinical studies were performed on the basis of the above-cited approaches, the outcomes were insufficient in terms of the strength and longevity of the cellular immune responses. On the other hand, passive transfer of MUC1-specific antibodies did not affect the growth of MUC1 expressing, experimentally transplanted melanomas in a mouse model (Tempero *et al*, 1999). In this model, CD4^+^ T cells were critical for rejection of MUC1-positive tumours (Tempero *et al*, 1998; VanLith *et al*, 2002), whereas other studies suggest that CD8^+^ T cells are required for MUC1-specific immunity against MUC1-positive pancreatic carcinoma (Morikane *et al*, 2001).

Although *in vitro* detection of cytolytic activity does not always correlate with *in vivo* tumour rejection, the potential of the C595scFv-Fc-IL2 fusion protein to activate immunocompetent resting NK and preactivated T cells after binding to MUC1-positive tumour cells is clearly demonstrated. Suppressive effects of the tumour environment, including locally accumulated suppressive cytokines, and other stromal elements may additionally modulate the efficacy of the fusion protein *in vivo*. The fusion protein, however, provides a specific tool to increase stimulatory signals to both activated T and resting NK cells in the vicinity of MUC1-positive tumour cells in order to improve the antitumour immune response.
